# Comparison of Fractures Among Older Adults Who Are Ambulatory vs Those Who Use Wheelchairs in Sweden

**DOI:** 10.1001/jamanetworkopen.2022.55645

**Published:** 2023-02-13

**Authors:** Kristian F. Axelsson, Henrik Litsne, Mattias Lorentzon

**Affiliations:** 1 Sahlgrenska Osteoporosis Centre, Institute of Medicine, Sahlgrenska Academy, University of Gothenburg, Gothenburg, Sweden; 2 Region Västra Götaland, Närhälsan Norrmalm Health Centre, Skövde, Sweden; 3 Mary McKillop Institute for Health Research, Australian Catholic University, Melbourne; 4 Region Västra Götaland, Geriatric Medicine, Sahlgrenska University Hospital, Mölndal, Sweden

## Abstract

**Importance:**

Several diseases and conditions, such as cerebrovascular disease, arthritis, previous fractures, neurological diseases, or amputation, can result in severe immobility justifying wheelchair use for increased mobility. Immobility results in disuse osteoporosis and is considered a risk factor for fracture, although there are no large cohort studies that have investigated fracture risk in patients who use wheelchairs compared with an ambulatory control group.

**Objective:**

To investigate whether immobilized adults who used wheelchairs had a different risk of fracture and injurious falls compared with matched ambulatory controls.

**Design, Setting, and Participants:**

This retrospective cohort study compared patients who used wheelchairs and controls (propensity score matched 1:1 using 22 variables relating to anthropometrics, general condition, comorbidity, and fall and fracture risk), identified through a national database of adults 65 years or older who underwent a health evaluation (baseline) at Swedish health care facilities. Patients were followed up from January 1, 2007, to December 31, 2017, and data analysis was performed between June 1 and 30, 2022.

**Main Outcomes and Measures:**

Incident fracture, injurious falls without fracture, and deaths.

**Results:**

A total of 55 442 adults using wheelchairs were included in the analysis (mean [SD] age, 83.2 [8.3] years; 60.5% women). Those who used wheelchairs and the 55 442 matched controls were followed up for a median of 2.0 (IQR, 0.5-3.2) and 2.3 (IQR, 0.8-3.6) years, respectively. Patients who used wheelchairs had a lower risk of any fracture (hazard ratio [HR], 0.43 [95% CI, 0.41-0.44]), major osteoporotic fracture (HR, 0.32 [95% CI, 0.31-0.33]), and hip fracture (HR, 0.30 [95% CI, 0.28-0.32]) compared with the ambulatory controls, associations that were only marginally affected by multivariable (same as the matching variables) adjustment. The risk of fall injury was lower among those who used wheelchairs than among ambulatory controls (unadjusted HR for Cox proportional hazards models, 0.48 [95% CI, 0.47-0.50]) and remained highly similar after adjustments. Patients who used wheelchairs had a significantly increased risk of death (HR, 1.35 [95% CI, 1.33-1.36]) compared with controls. Association between wheelchair use and fracture outcomes and injurious falls, calculated using a Fine and Gray model with death as a competing risk, was similar to associations obtained using Cox proportional hazards regression for all fracture outcomes.

**Conclusions and Relevance:**

In this retrospective cohort study of older adults, wheelchair use was associated with a lower risk of fracture than observed in ambulatory controls. These findings suggest that immobility associated with wheelchair use should not be considered a risk factor for fracture.

## Key Points

**Question:**

Do older adults who use wheelchairs have a different fracture risk than ambulatory adults?

**Findings:**

This cohort study of 55 442 adults in Sweden found that wheelchair use was associated with a reduced risk of any fracture, major osteoporotic fracture, and hip fracture compared with 55 442 ambulatory controls. A similarly lower risk was observed for injurious falls without fracture, suggesting that the observed lower fracture risk is at least partly due to fewer falls among adults using wheelchairs.

**Meaning:**

These findings suggest that immobility associated with wheelchair use should not be considered a risk factor for fracture.

## Teaser

This nationwide cohort study investigates whether immobilized patients who use wheelchairs have a different risk of fracture and injurious falls compared with a matched control group of ambulatory adults in Sweden.

## Introduction

Wheelchair use can facilitate activities of daily living and increase mobility and independence and has been associated with a better quality of life in older adults with impaired physical function.^1,2^ Several diseases and conditions, such as cerebrovascular disease, arthritis, previous fractures, neurological diseases, chronic obstructive pulmonary disease, or amputation, can result in severe immobility justifying wheelchair use for increased mobility.^3^ More than 15 million Americans 65 years or older struggle with ambulatory activities, and approximately 1.5% of them use wheelchairs.^4^

Immobility per se also results in increased risk of a multitude of negative outcomes, including a higher risk of thromboembolic events, pressure ulcers, and bone loss.^3,4,5,6,7^ Absence of skeletal weight loading (eg, immobility after a stroke, prolonged bed rest, or loss of gravity during spaceflight) leads to rapid and substantial loss of bone mineral density (BMD), reaching approximately 1% per month and resulting in disuse osteoporosis.^8,9^ Normally, weight loaded bones of the lower extremities are the most sensitive to bone loss following immobility. In a cohort of patients with spinal cord injury, the loss of bone mass was the highest (52%) at the distal femur and proximal tibia (70%), bone sites most commonly affected by fracture among patients with spinal cord injuries.^10,11^ The mechanism behind this effect involves lack of skeletal compression causing reduced canalicular fluid, which leads to hypoxia among the osteocytes and failure to transduce sufficient mechanical stimuli, thereby increasing osteoclastic activity, resulting in bone resorption.^7^ Both falls during transfers to and from the wheelchair and bone loss at these skeletal sites likely contribute to the increased risk of fracture in the lower extremities observed in patients with immobility.^12^

Immobility is considered a risk factor for secondary osteoporosis and fracture in many clinical guidelines globally, and physicians are encouraged to evaluate fracture risk and presence of osteoporosis in these patients.^13,14,15^ While the loss of bone mass due to lack of mobility and skeletal loading is intuitive and well documented, there is very limited evidence regarding the fracture risk in patients using wheelchairs to support evaluation and treatment of patients using wheelchairs with osteoporotic medications. Several small studies in a scoping review^16^ have found an increased risk of lower extremity fractures in patients with spinal cord injury, and a recent large prospective study^17^ found that the risk of fractures after stroke was higher in patients who had severe disability, although the proportion of these patients using wheelchairs was not reported. Despite the risk of fractures when transferring to or from the wheelchair, patients who use wheelchairs are likely protected from falling. Thus, immobilization leading to wheelchair use may not be associated with an increased risk of falls and fractures. A potential protective effect of wheelchair use on these outcomes may be clinically important and provide guidance regarding the need for evaluation of fracture risk and osteoporosis. The primary aim of the present study was to investigate whether patients with immobility who use wheelchairs have a different risk of fracture and injurious falls compared with a matched control group of ambulatory adults in a large nationwide Swedish cohort.

## Methods

### Study Design

This retrospective cohort study used national registers in Sweden to compare the risk of fractures between adults who used wheelchairs and propensity score–matched ambulatory controls. The study was approved by the Swedish Ethical Review Authority, which issued a waiver of the patient informed consent requirement because all the data used were collected from registers without the investigators having direct contact with participants. We followed the Strengthening the Reporting of Observational Studies in Epidemiology (STROBE) reporting guideline.

### Data Sources

The Senior Alert register was designed to follow and support improvements in preventive care for older adults. Data were registered by health care professionals in connection with a health care visit. The register covered more than 90% of all municipalities and counties in Sweden^18^ and included information on mobility categorized as (1) ambulatory with or without aid, (2) ambulatory with the help of staff, (3) full-day use of a wheelchair, or (4) bedridden. Patients in categories 1 and 3 were used in subsequent analyses. Data on weight, height, general condition, and fluid and food intake were also available in the Senior Alert register. Information regarding comorbidities, falls, and fractures were retrieved from the National Patient Register, including hospital-based diagnoses from both inpatient and outpatient visits. Socioeconomic data were retrieved from Statistics Sweden, and the date of death was retrieved from the Swedish Cause of Death Register. The Swedish Prescribed Drug Register, starting July 1, 2005, was used to collect data on medications. All inhabitants in Sweden are assigned a personal identification number at birth or at the time of immigration, enabling linkage between the registers.

### Study Population

All men and women 65 years or older included in the Senior Alert register between January 1, 2007, and December 31, 2017, either those using a wheelchair or those who were ambulatory controls, were eligible for this study (eFigure 1 in Supplement 1). Patients with extreme entries regarding height, weight, or body mass index (top and bottom 0.05%) were excluded because of probable register entry errors. The first date of registration in Senior Alert was used as baseline.

### Outcomes

Fracture outcomes, fall injuries, and deaths were assessed. Any fracture included all nonpathological fracture diagnoses regardless of type of trauma (head and phalangeal fractures excluded). Major osteoporotic fractures (MOF) included the hip, vertebrae, proximal humerus, wrist, and pelvis. Hip fracture included fractures of the femoral head, neck, trochanter, or subtrochanteric part of the femur accompanied with a code for a surgical procedure. Specific fracture types were also defined and assessed, including vertebral, distal femoral, proximal tibia, ankle, proximal humerus, and wrist. Injurious falls were defined as any hospital event with a code for injury and fall but without a fracture code (eTable 1 in Supplement 1).

### Baseline Data

Many covariates representing prevalent illnesses and prescribed medications with potential impact on a patient’s comorbidity and risk of fracture were selected as matching variables (Table 1). The Charlson Comorbidity Index was calculated to summarize and quantify comorbidity.^19^ The FRAX (Fracture Risk Assessment Tool) 10-year probabilities for hip fracture and MOF without BMD were calculated using age, sex, weight, height, previous fracture, oral glucocorticoid use, rheumatoid arthritis, secondary osteoporosis, and a register-based proxy variable for high alcohol intake (alcohol-related disease) (eTable 2 in Supplement 1), using the Sweden-specific FRAX model.^20^ Data on smoking and parental hip fracture were missing. These risk factors were assumed to be absent. Prevalent medication variables included the last 12 months of prescriptions from both hospitals and primary care practices. Also, several conditions possibly associated with wheelchair use were defined but not used as matching variables (eTable 3 in Supplement 1).

**Table 1.** Baseline Patient Characteristics

Variables used in matching	Patient group^a^				
	Wheelchair use (n = 55 442)	Ambulatory control			
		Before propensity score matching (n = 410 299)	SMD^b^	After propensity score matching (n = 55 442)	SMD^b^
Age, mean (SD), y	83.2 (8.3)	80.1 (8.1)	0.38	83.3 (8.1)	0.02
Sex					
Men	21 895 (39.5)	183 702 (44.8)	0.11	21 736 (39.2)	0.01
Women	33 547 (60.5)	226 597 (55.2)		33 706 (60.8)	
Weight, mean (SD), kg	68.8 (17.0)	72.3 (16.0)	0.21	68.7 (16.3)	0.01
Height, mean (SD), cm	166.3 (10.3)	167.2 (9.8)	0.09	166.1 (10.0)	0.02
Patient registration site					
Nursing home	34 773 (62.7)	90 275 (22.0)	0.90	34 814 (62.8)	0.01
Hospital	17 354 (31.3)	270 303 (65.9)		17 174 (31.0)	
Private residence	3315 (6.0)	49 721 (12.1)		3454 (6.2)	
General condition					
Good	16 071 (29.0)	200 992 (49.0)	0.47	16 187 (29.2)	0.05
Fairly good	30 856 (55.7)	185 437 (45.2)		31 704 (57.2)	
Poor	7764 (14.0)	21 569 (5.3)		6854 (12.4)	
Very poor	751 (1.4)	2301 (0.6)		697 (1.3)	
Fluid intake, mL/d^c^					
>1000	24 733 (44.6)	257 539 (62.8)	0.40	24 920 (44.9)	0.03
700-1000	21 809 (39.3)	122 862 (29.9)		22 212 (40.1)	
500-700	7531 (13.6)	26 094 (6.4)		7061 (12.7)	
<500	1369 (2.5)	3804 (0.9)		1249 (2.3)	
Food intake, portion size, %					
100 (normal)	29 632 (53.4)	293 174 (71.5)	0.39	29 788 (53.7)	0.02
75	11 557 (20.8)	59 316 (14.5)		11 824 (21.3)	
50	10 037 (18.1)	43 909 (10.7)		9925 (17.9)	
<50	4216 (7.6)	13 900 (3.4)		3905 (7.0)	
Sickness benefits	185 (0.3)	2726 (0.7)	0.05	205 (0.4)	0.01
Marital status					
Widowed	24 317 (43.9)	150 322 (36.6)	0.21	24 828 (44.8)	0.02
Married	16 787 (30.3)	163 037 (39.7)		16 275 (29.4)	
Divorced	8215 (14.8)	61 536 (15.0)		8198 (14.8)	
Unmarried	6123 (11.0)	35 404 (8.6)		6141 (11.1)	
Urban residency, population >200/km^2^	11 776 (21.2)	41 371 (10.1)	0.31	11 659 (21.0)	0.01
Non-Nordic citizenship at birth	2353 (4.2)	15 513 (3.8)	0.02	2323 (4.2)	0.003
Charlson Comorbidity Index					
Mean (SD)	2.26 (2.16)	1.88 (2.11)	0.18	2.25 (2.39)	0.002
0	11 857 (21.4)	122 677 (29.9)		13 592 (24.5)	
1	12 957 (23.4)	93 292 (22.7)		12 364 (22.3)	
2	10 262 (18.5)	83 688 (20.4)		10 832 (19.5)	
≥3	20 366 (36.7)	110 642 (27.0)		18 654 (33.6)	
Osteoporosis diagnosis	3676 (6.6)	18 139 (4.4)	0.10	3729 (6.7)	0.004
Conditions associated with osteoporosis^d^	3959 (7.1)	19 973 (4.9)	0.10	3946 (7.1)	0.001
Alcohol-related disease	1325 (2.4)	8162 (2.0)	0.03	1421 (2.6)	0.01
Rheumatoid arthritis	1687 (3.0)	9730 (2.4)	0.04	1693 (3.1)	0.001
Prevalent fracture	26 982 (48.7)	120 877 (29.5)	0.40	27 237 (49.1)	0.01
Prevalent fall injury	16 800 (30.3)	76 871 (18.7)	0.27	16 896 (30.5)	0.004
Osteoporosis medication use	3397 (6.1)	21 944 (5.3)	0.03	3474 (6.3)	0.01
Calcium plus vitamin D supplementation	6906 (12.5)	41 782 (10.2)	0.07	7198 (13.0)	0.02
Oral prednisolone use	7097 (12.8)	55 285 (13.5)	0.02	7027 (12.7)	0.004
FRAX score, mean (SD), %^e^					
MOF	25.3 (12.6)	21.3 (11.9)	0.35	25.6 (12.8)	0.02
Hip	14.7 (9.5)	11.5 (8.4)	0.36	14.8 (9.5)	0.01

Abbreviations: FRAX, Fracture Risk Assessment Tool; MOF, major osteoporotic fractures; SMD, standardized mean differences.

^a^ Unless otherwise indicated, data are expressed as No. (%) of patients. Percentages have been rounded and may not total 100. The historic window was since 1998 for fracture and fall, 5 years for other diagnoses, and 1 year for medications. Detailed definitions of selected covariables are provided in eTable 2 in Supplement 1.

^b^ See eMethods in Supplement 1 for formulas used to calculate.

^c^ The form used to record fluid intake in the Senior Alert Register uses these numbers, allowing overlap.

^d^ Includes hyperthyroidism, hypogonadism, malnutrition, osteogenesis imperfecta, chronic liver disease, and hyperparathyroidism.

^e^ Scores indicate 10-year probabilities (without bone mineral density) for hip fracture and MOF.

### Statistical Analysis

Statistical analyses were performed between June 1 and 30, 2022. Each patient who used a wheelchair was matched to an ambulatory control using 1:1 multivariable propensity score matching.^21^ Descriptive baseline statistics before and after matching are presented in terms of counts with percentage for categorical variables, means with SDs for normally distributed continuous variables, and medians with IQRs for other continuous variables. Standardized mean differences were calculated to present differences in baseline characteristics between those who used wheelchairs and controls. Event rates were calculated as the number of events per 1000 person-years and are presented with exact Poisson 95% CIs. The cumulative incidence of events was estimated using 1 minus the Kaplan-Meier estimate of the corresponding survival function and presented with 95% CIs.

Cox proportional hazards regression models were used to calculate hazard ratios (HRs), both unadjusted with wheelchair user or ambulatory status as the only independent variable as well as with extensive multivariable adjustment (variables listed in Table 1). The follow-up time was censored for the end of study (December 31, 2017), emigration, and death. The Cox assumption of proportional hazards regression was tested using graphical methods. Interactions were tested using fully adjusted Cox proportional hazards regression models, with interaction terms for the group variable (wheelchair use) and sex and age. For analysis of interaction, 2-sided *P* values of less than .10 were considered significant. As a sensitivity analysis, all the nonmatched controls were used in a fully adjusted Cox proportional hazards regression model. Also, to assess whether the risk varied depending on the potential underlying cause of wheelchair use, possible diagnoses for conditions associated with wheelchair use were defined and identified, which allowed categorization of patients who used wheelchairs into subgroups, which were then compared categorically with the ambulatory matched controls.

To assess the potential impact of death as a competing risk, the cumulative incidence or subdistribution function of fracture with death as a competing risk was estimated using the Aalen-Johansen estimator.^22^ Also, for a subset of 30 000 randomly selected persons, the subdistribution hazard for fracture was compared between patients who used wheelchairs and ambulatory controls using a Fine and Gray model with death as the competing risk.^23^ Statistical analyses were performed using R, version 4.02, and R Studio, version 1.4.1106 (R Program for Statistical Computing).

## Results

### Study Population

A total of 55 442 patients who used wheelchairs were included in the analysis. The mean (SD) age was 83.2 (8.3) years; 60.5% of patients were women and 39.5% were men. An equal number of matched controls were included. There were no large differences between those who used wheelchairs and the matched controls in terms of the baseline characteristics used in the matching. After matching, differences were 0.05 or less (Table 1). For example, the mean 10-year FRAX probability of MOF was 25.3% (12.6%) among the patients who used wheelchairs, compared with 25.6% (12.8%) among the ambulatory controls. Among those who used wheelchairs, it was more common with stroke, previous femur fracture, previous lower leg fracture, hemiplegia, paraplegia or tetraplegia, epilepsy, Parkinson disease, and spinal cord injury (eTable 3 in Supplement 1). Patients who used wheelchairs and matched controls were followed up for a median of 2.0 (IQR 0.5-3.2) and 2.3 (IQR 0.8-3.6) years, respectively.

### Risk of Fractures

**Figure 1.**
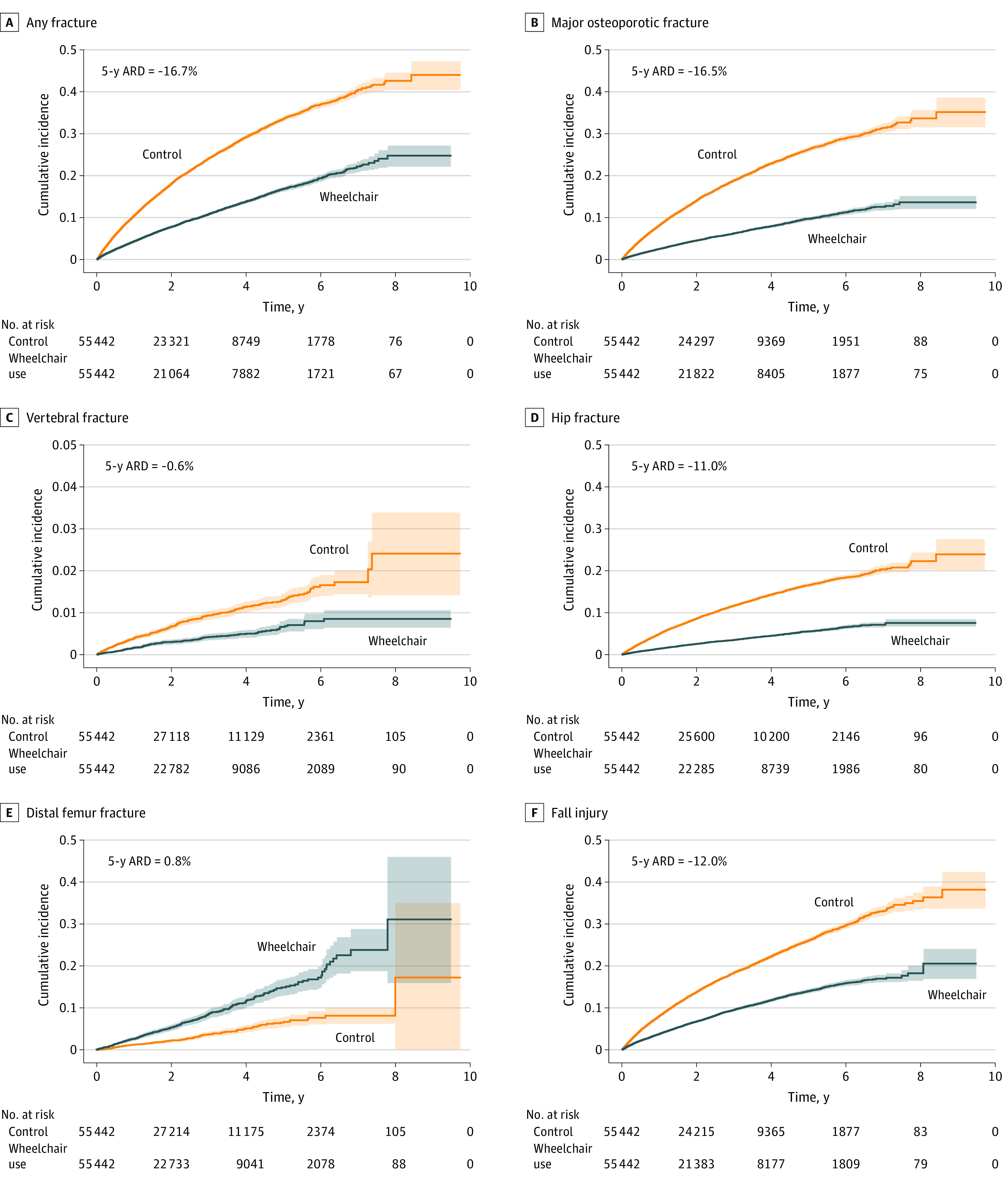
Cumulative Incidences for Patients Who Used Wheelchairs vs Matched Ambulatory Controls The cumulative incidence of events was estimated using 1 minus the Kaplan-Meier estimate of the corresponding survival function and presented with 95% CIs. Fall injury indicated all fall-related injuries not resulting in a fracture. The 5-year absolute risk difference (ARD) noted in the graph derives from the curve.

During follow-up, 4148 patients who used wheelchairs (7.5%) and 10 344 ambulatory controls (18.7%) sustained any fracture, translating to incidence rates of 39.3 (95% CI, 38.1-40.5) and 91.2 (95% CI, 89.4-93.0) per 1000 person-years, respectively. The patients who used wheelchairs had a 2.3-fold reduced risk of any fracture (HR, 0.43 [95% CI, 0.41-0.44]), 3.1-fold reduced risk of MOF (HR, 0.32 [95% CI, 0.31-0.33]), and 3.3-fold reduced risk of hip fracture (HR, 0.30 [95% CI, 0.28-0.32]) compared with ambulatory controls. The risks of vertebral fracture (HR, 0.45 [95% CI, 0.37-0.54]), proximal humerus fracture (HR, 0.42 [95% CI, 0.38-0.47]), and wrist fracture (HR, 0.23 [95% CI, 0.20-0.26]) were also substantially reduced compared with the ambulatory controls (Figure 1, Table 2, and eTable 4 in Supplement 1), associations that changed only marginally after multivariable adjustment. In contrast, patients who used wheelchairs had a 2.4-fold increased risk of distal femur fracture (HR, 2.37 [95% CI, 1.96-2.86]) and a 1.6-fold risk of proximal tibia fracture (HR, 1.62 [95% CI, 1.31-2.00]). When comparing those who used wheelchairs with the unmatched controls, the associations were similar (eTable 5 in Supplement 1).

**Table 2.** Outcomes for Patients Who Used Wheelchairs vs Matched Ambulatory Controls

Outcome^a^	Patient group	
	Propensity score–matched ambulatory control^b^ (n = 55 442)	Wheelchair use^c^ (n = 55 442)
**Any fracture**		
No. (%) of patients	10 344 (18.7)	4148 (7.5)
Rate per 1000 person-years (95% CI)	91.2 (89.4-93.0)	39.3 (38.1-40.5)
HR (95% CI)		
Unadjusted	1 [Reference]	0.43 (0.41-0.44)
Adjusted	1 [Reference]	0.43 (0.42-0.45)
**Major osteoporotic fracture**		
No. (%) of patients	8046 (14.5)	2399 (4.3)
Rate per 1000 person-years (95% CI)	68.5 (67.0-70.0)	22.0 (21.2-22.9)
HR (95% CI)		
Unadjusted	1 [Reference]	0.32 (0.31-0.33)
Adjusted	1 [Reference]	0.33 (0.31-0.34)
**Vertebral fracture**		
No. (%) of patients	392 (0.7)	157 (0.3)
Rate per 1000 person-years (95% CI)	3.04 (2.75-3.36)	1.39 (1.18-1.62)
HR (95% CI)		
Unadjusted	1 [Reference]	0.45 (0.37-0.54)
Adjusted	1 [Reference]	0.45 (0.37-0.54)
**Hip fracture**		
No. (%) of patients	4971 (9.0)	1363 (2.5)
Rate per 1000 person-years (95% CI)	40.5 (39.4-41.7)	12.3 (11.7-13.0)
HR (95% CI)		
Unadjusted	1 [Reference]	0.30 (0.28-0.32)
Adjusted	1 [Reference]	0.31 (0.29-0.33)
**Distal femur fracture**		
No. (%) of patients	159 (0.3)	327 (0.6)
Rate per 1000 person-years (95% CI)	1.23 (1.05-1.44)	2.90 (2.59-3.23)
HR (95% CI)		
Unadjusted	1 [Reference]	2.37 (1.96-2.86)
Adjusted	1 [Reference]	2.53 (2.09-3.06)
**Proximal tibia fracture**		
No. (%) of patients	145 (0.3)	205 (0.4)
Rate per 1000 person-years (95% CI)	1.12 (0.947-1.32)	1.82 (1.58-2.08)
HR (95% CI)		
Unadjusted	1 [Reference]	1.62 (1.31-2.00)
Adjusted	1 [Reference]	1.61 (1.30-1.99)
**Fall injury without fracture**		
No. (%) of patients	7930 (14.3)	3544 (6.4)
Rate per 1000 person-years (95% CI)	67.8 (66.3-69.3)	33.1 (32.0-34.2)
HR (95% CI)		
Unadjusted	1 [Reference]	0.48 (0.47-0.50)
Adjusted	1 [Reference]	0.49 (0.47-0.51)
**Death**		
No. (%) of patients	34 279 (61.8)	40 722 (73.4)
Rate per 1000 person-years (95% CI)	265 (262-268)	360 (356-363)
HR (95% CI)		
Unadjusted	1 [Reference]	1.35 (1.33-1.36)
Adjusted	1 [Reference]	1.40 (1.38-1.42)

Abbreviation: HR, hazard ratio.

^a^ Event rates were calculated as the number of events per 1000 person-years and are presented with exact Poisson 95% CIs. The adjusted Cox proportional hazards model is adjusted for age, sex, weight, height, patient place at time of registration, general condition, fluid intake, food intake, sickness benefits, marital status, urban residency, non-Nordic citizenship at birth, Charlson Comorbidity Index, osteoporosis diagnosis, conditions associated with osteoporosis, alcohol-related disease, rheumatoid arthritis, prevalent fracture, prevalent fall injury, osteoporosis medication use, calcium plus vitamin D supplementation, and oral prednisolone use. *P* < .001 for all comparisons.

^b^ Median time at risk, 2.3 (IQR, 0.8-3.6) years.

^c^ Median time at risk, 2.0 (IQR, 0.5-3.2) years.

### Risk of Fractures per Diagnosis Subgroup, Age, Sex, and Inclusion Site

**Figure 2.**
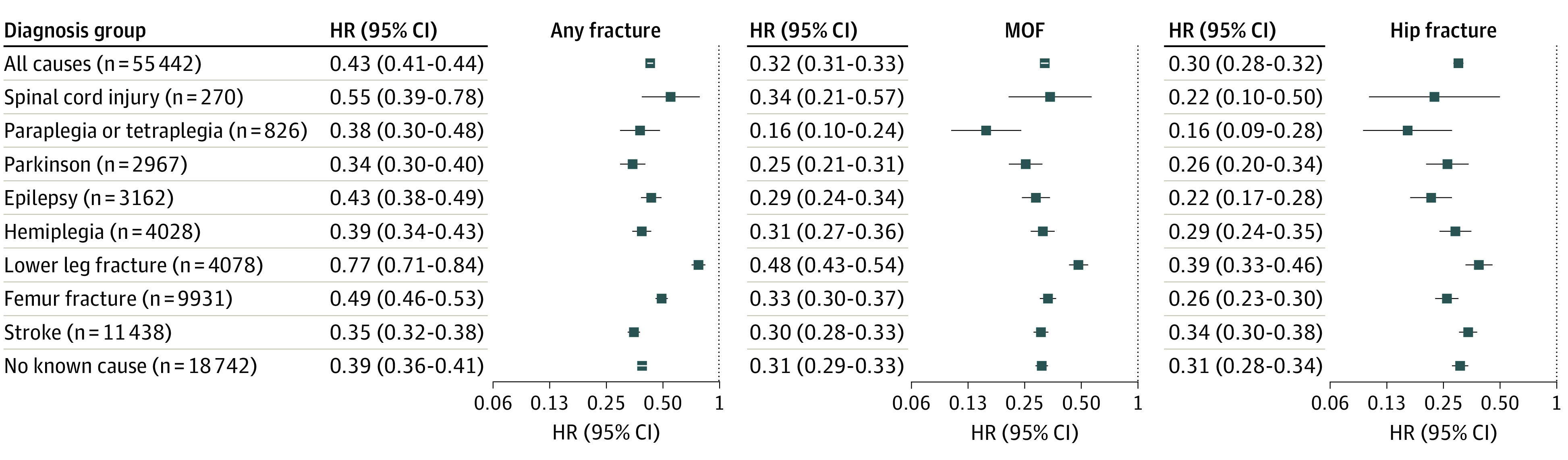
Risk of Fracture per Diagnosis Subgroup Among Patients Who Used Wheelchairs vs Matched Ambulatory Controls Relative risk of any fracture, major osteoporotic fracture (MOF), and hip fracture in patients who used wheelchairs was compared with matched ambulatory controls using unadjusted Cox proportional hazards models by subgroup (underlying diagnosis associated with immobility) as a categoric variable, with ambulatory controls used as the reference group. Corresponding number of events, event ratios, and adjusted hazard ratios (HRs) are presented in eTable 6 in Supplement 1. Multiple underlying diagnoses were possible, but each patient who used a wheelchair was only included once and assigned to the smallest group. *P* < .001 for interaction for all 3 outcomes.

**Figure 3.**
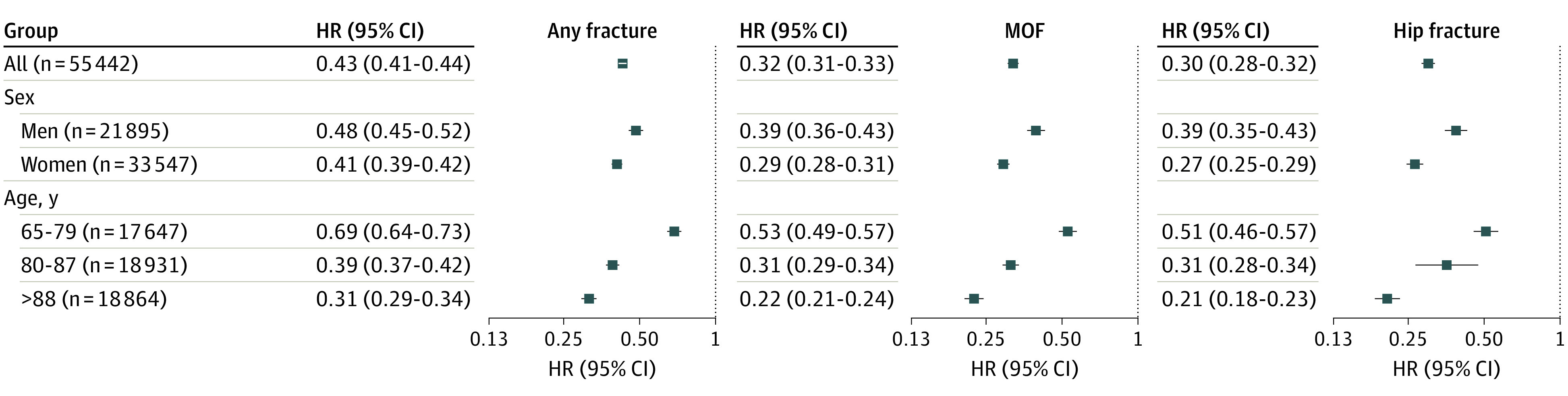
Risk of Fracture per Sex and Age Group Among Patients Who Used Wheelchairs vs Matched Ambulatory Controls Relative risk of any fracture, major osteoporotic fracture (MOF), and hip fracture in patients who used wheelchairs was compared with matched ambulatory controls using unadjusted Cox proportional hazards regression models. Corresponding number of events, event ratios, and adjusted hazard ratios (HRs) are presented in eTables 7 and 8 in Supplement 1. *P* < .001 for interaction for all 3 investigated fracture outcomes.

Compared with ambulatory controls, the risk of fracture was consistently lower among patients who used wheelchairs regardless of diagnosis group (Figure 2) and was only marginally affected by multivariable adjustment (eTable 6 in Supplement 1). There were significant interactions between the group variable and sex and age (Figure 3 and eTables 7 and 8 in Supplement 1). For hip fracture, MOF, and any fracture, the differences in risk between the group using wheelchairs and controls were greater among women than among men. For all these fracture outcomes, the risk difference between patients who used wheelchairs and controls was greater with increasing age (eg, >15% for all falls among those 88 years or older vs 4.4% for those aged 65-79 years). Differences in fracture risk between those who used wheelchairs and controls were consistent according to inclusion site (nursing homes, hospitals, and private residences) (eTable 9 in Supplement 1).

### Risk of Injurious Falls Without Fracture

The risk of injurious falls without fracture was 2.1-fold lower among patients who used wheelchairs than among ambulatory controls (HR for unadjusted Cox proportional hazards models, 0.48 [95% CI, 0.47-0.50]). The risk remained similar after adjustments (Table 2).

### Mortality and Competing Risk

There were 40 722 deaths (73.4%) among the patients who used wheelchairs during follow-up compared with 34 279 (61.8%) among the ambulatory controls, translating to incidence rates of 360 (95% CI, 356-363) and 265 (95% CI, 262-268) per 1000 person-years, respectively. Mortality rates were in general lower in both groups among those from private residences and hospitals than from nursing homes (eTable 9 in Supplement 1). Mortality rates in both groups increased with age span (eTable 8 in Supplement 1). Patients who used wheelchairs had a significantly increased risk of death (HR, 1.35 [95% CI, 1.33-1.36]) compared with controls, an association slightly attenuated by multivariable adjustment (Table 2). The difference between the patients who used wheelchairs and the control group increased with increasing age, both in terms of absolute and relative risk of death (eTable 8 in Supplement 1). Visualization of the cumulative incidence functions of each outcome with death as a competing risk revealed a minimal impact on the studied associations (eFigure 2 in Supplement 1). Subdistribution HRs for the association among wheelchair use, fracture outcomes, and injurious falls, calculated using a Fine and Gray model with death as a competing risk, were similar to HRs obtained using Cox proportional hazards regression, both for the whole cohort and for all age groups analyzed separately (eTable 10 in Supplement 1).

## Discussion

In this nationwide cohort study of older adults who used wheelchairs and propensity score–matched ambulatory controls investigated from 2007 to 2017, those who used wheelchairs had a substantially lower risk of any fracture (2.3-fold), MOF (3.1-fold), and hip fracture (3.3-fold). A similarly lower risk was observed for injurious falls without fracture (2.1-fold), suggesting that the observed lower fracture risk is at least partly due to fewer falls in those using wheelchairs. As expected, patients who used wheelchairs had higher mortality than ambulatory controls, but adjusting for the competing risk of death did not materially change the associations between wheelchair use and fracture. These results provide support for physicians who consider recommending wheelchair use for older frail adults with a very high fracture risk.

Mechanical loading is required for maintaining skeletal strength and integrity. Loss of weight loading leads to disuse osteoporosis, seen after spinal cord injury, stroke, prolonged bed rest, spaceflight, or neurological disease.^7,8,10^ Bone loss is known to be the greatest in the first 2 years following the loss of weight loading, with a subsequent period of stable bone metabolism.^24^ Although the concept of disuse osteoporosis is well established, only limited evidence is available regarding the effect of conditions associated with disuse osteoporosis on fracture risk, which in this context is the most important clinical consequence. As reported from a questionnaire-based, retrospective case and control study, 438 patients with spinal cord injury had a higher fracture risk than controls, an association only found after their injury, but the observation time was limited. Data on fractures were self-reported, which is known to be more prone to errors than fractures ascertained using radiography or medical records.^25^ Furthermore, the mean age of the patients was only 42 years, substantially younger than that of patients using wheelchairs in the present analysis. Thus, the risk of fracture in older patients with spinal cord injury many years ago has not been investigated. In the present study, all patients using wheelchairs, including those with previous spinal cord injury, had a substantially lower fracture risk as well as a lower risk of other injurious falls, than ambulatory controls, indicating that wheelchair use reduces falls and that this possible effect outweighs any developed disuse osteoporosis, also in those with spinal cord injury.

Despite the accepted notion that immobility causes osteoporosis and should be considered an important clinical risk factor for fracture,^13,14,15^ no large, well-controlled studies investigating fracture risk in adults dependent on wheelchairs have been performed. Recently, a small study (n = 261) on institutionalized adults with epilepsy and intellectual disability^26^ found that the risk of any fracture was 68% lower in patients dependent on wheelchairs than in those able to walk, although this finding was based on very few patients dependent on wheelchairs (n = 58) and a low number of fractures (n = 14). In a study of 2711 nursing home residents, fully ambulatory residents did not have a significantly higher fracture risk (odds ratio, 1.25 [95% CI, 0.71-2.20]) than those who used wheelchairs, but that analysis was limited in that residents were only observed for 1 year, time to event was not considered, few residents experienced a fracture (n = 165), and those with prior hip fracture were excluded, factors which likely affected the results.^27^ In contrast, the present analysis demonstrated that the lower risk associated with wheelchair use for all types of investigated fractures outcomes was most pronounced in the subgroup of nursing home residents. The risk differences were smaller for patients included at hospitals, which could be because the underlying condition may more frequently be reversible.

In this analysis, the mortality rate was considerable in both groups, but adjustment for competing risk of death did not materially change the associations between wheelchair use and fracture risk. Furthermore, the lower risk of fracture in patients who used wheelchairs was consistent across age groups, being present also in the youngest age group with a substantially lower mortality, supporting a mortality-independent association between wheelchair use and fracture risk.

As observed in patients with disuse osteoporosis, the risk of distal femur fracture was increased by over 2.4-fold in patients who used wheelchairs, consistent with previous studies in patients with spinal cord injury.^28,29^ In agreement with previous studies, the risk of proximal tibia fracture was increased by nearly 2-fold in the present analyses. We hypothesize that the increased risk of knee fractures may be due to a combination of disuse osteoporosis known to affect the lower limbs, and that the distal femur and proximal tibia are skeletal sites being more exposed when positioned in a wheelchair than other skeletal sites. However, it should be emphasized that the absolute risk (0.6% and 0.4%, respectively, during follow-up) for this type of fracture in patients who used wheelchairs was very low compared with the risk of any fracture and MOF, experienced by 18.7% and 14.5% of the ambulatory controls, respectively, during follow-up. Thus, in terms of the overall fracture burden, wheelchair use was associated with a considerable benefit.

Although continued regular physical exercise maintains physical fitness and is associated with reduced risk of thromboembolic events, pressure ulcers, and bone loss,^3,4,5,6,7^ increasing frailty and falls risk in an older frail patient could at some point justify for the clinician to deliberate the option of prescribing a wheelchair to increase patient mobility and at the same time reduce the risk of falls and fractures. The results from this study indicate that the risk of fall and fracture is decreased in patients who use wheelchairs, supporting the prescription of this aid if the risk of falls and fractures is deemed high. It should, however, be acknowledged that this study does not factor in potentially other negative outcomes associated with wheelchair use.

An apparent interaction between age and the group variable (wheelchair use vs ambulatory) was observed for all fracture outcomes in our analyses. In contrast to most other risk factors for fracture, such as prevalent fracture or parental hip fracture,^30,31^ the risk difference between patients who used wheelchairs and ambulatory controls rose with increasing age. The absolute risk differences exceeded 15% for any fracture in those 88 years or older, as opposed to a risk difference of 4.4% in those aged 65 to 79 years. Thus, these data indicate that wheelchair use reduces fracture risk in the age group with the highest absolute fracture risk, which implies a substantial clinical benefit in terms of lowering fracture numbers in the oldest patients.

### Strengths and Limitations

This study has several strengths. To our knowledge, it is the by far largest cohort study investigating the risk of fractures and injurious falls among patients who use wheelchairs and ambulatory controls, allowing investigation with adequate statistical power of more rare outcomes, such as hip fractures and lower extremity fractures. Using several data sources that provided access to anthropometrics, patient evaluations, diagnoses, medications, and surgical procedures, detailed matching according to many important comorbidities and risk factors provided a balanced control group. This in combination with additional statistical adjustment for covariates enabled investigation of associations with minimal bias.

The study also has limitations. First, due to the observational design, causality cannot be determined. Second, the specific diagnosis or condition that led to the wheelchair use was not available, although diagnoses indicative of the underlying reason for the immobility leading to wheelchair use were available. Third, data on BMD were not available. Fourth, the duration of wheelchair use was unknown and is likely an important factor for the severity of bone loss and fracture risk.^10,32^ However, risk differences between patients who used wheelchairs and controls remained over time for fracture outcomes. While the data did not allow comparison of long-term and short-term wheelchair use, the findings of subgroup analyses indicate that the risk of fracture in conditions associated with short-term use (eg, lower leg fracture) was less reduced than in conditions associated with long-term use (eg, stroke and neurological conditions).

## Conclusions

The findings of this cohort study suggest that wheelchair-induced immobility was associated with a substantially reduced risk of fracture, indicating that wheelchair use, especially in older adults, offers fall protection that seems to surpass any adverse effects of disuse osteoporosis. These results may aid physicians’ decision-making when considering prescribing wheelchair use to older patients who have a disability.

## Notes

Supplement 1. **eMethods.** Additional Information on Registers Used and Standardized Mean Difference **eFigure 1.** Study Population **eTable 1.** Detailed Definitions of Outcomes **eTable 2.** Detailed Definitions of Selected Covariates **eTable 3.** Baseline Characteristics of Variables Possibly Associated With Wheelchair Use **eTable 4.** Other Outcomes for Patients Who Used Wheelchairs vs Matched Ambulatory Controls **eTable 5.** Outcomes for Patients Who Used Wheelchairs vs Unmatched Ambulatory Controls **eTable 6.** Outcomes for Patients Who Used Wheelchairs vs Matched Ambulatory Controls per Subgroup **eTable 7.** Outcomes for Patients Who Used Wheelchairs vs Matched Ambulatory Controls per Sex **eTable 8.** Outcomes for Patients Who Used Wheelchairs vs Matched Ambulatory Controls per Age Group **eTable 9.** Outcomes for Patients Who Used Wheelchairs vs Matched Ambulatory Controls per Inclusion Site **eFigure 2.** Cumulative Incidence Function in Patients Who Used Wheelchairs vs Matched Ambulatory Controls **eTable 10.** Fine and Gray–Adjusted Subdistribution Hazard Ratios for Patients Who Used Wheelchairs vs Matched Ambulatory Controls With Consideration of Competing Risk of Death

Supplement 2.
Data Sharing Statement
