# Supplementary material for: Comparison of Fractures Among Older Adults Who Are Ambulatory vs Those Who Use Wheelchairs in Sweden
Source: JAMA Netw Open. 2023 Feb 13;6(2):e2255645. doi: 10.1001/jamanetworkopen.2022.55645 (PMC9926324; doi:10.1001/jamanetworkopen.2022.55645)
Supplement: Supplement 1. — eMethods. Additional Information on Registers Used and Standardized Mean Difference eFigure 1. Study Population eTable 1. Detailed Definitions of Outcomes eTable 2. Detailed Definitions of Selected Covariates eTable 3. Baseline Characteristics of Variables Possibly Associated With Wheelchair Use eTable 4. Other Outcomes for Patients Who Used Wheelchairs vs Matched Ambulatory Controls eTable 5. Outcomes for Patients Who Used Wheelchairs vs Unmatched Ambulatory Controls eTable 6. Outcomes for Patients Who Used Wheelchairs vs Matched Ambulatory Controls per Subgroup eTable 7. Outcomes for Patients Who Used Wheelchairs vs Matched Ambulatory Controls per Sex eTable 8. Outcomes for Patients Who Used Wheelchairs vs Matched Ambulatory Controls per Age Group eTable 9. Outcomes for Patients Who Used Wheelchairs vs Matched Ambulatory Controls per Inclusion Site eFigure 2. Cumulative Incidence Function in Patients Who Used Wheelchairs vs Matched Ambulatory Controls eTable 10. Fine and Gray–Adjusted Subdistribution Hazard Ratios for Patients Who Used Wheelchairs vs Matched Ambulatory Controls With Consideration of Competing Risk of Death [file jamanetwopen-e2255645-s001.pdf]

Supplementary Online Content

Axelsson KF, Litsne H, Lorentzon M. Comparison of fractures among older adults who are ambulatory vs those who use wheelchairs in Sweden. *JAMA Netw Open*. 2023;6(2):e2255645. doi:10.1001/jamanetworkopen.2022.55645

eMethods. Additional Information on Registers Used and Standardized Mean Difference
eFigure 1. Study Population
eTable 1. Detailed Definitions of Outcomes
eTable 2. Detailed Definitions of Selected Covariates
eTable 3. Baseline Characteristics of Variables Possibly Associated With Wheelchair Use
eTable 4. Other Outcomes for Patients Who Used Wheelchairs vs Matched Ambulatory Controls
eTable 5. Outcomes for Patients Who Used Wheelchairs vs Unmatched Ambulatory Controls
eTable 6. Outcomes for Patients Who Used Wheelchairs vs Matched Ambulatory Controls per Subgroup
eTable 7. Outcomes for Patients Who Used Wheelchairs vs Matched Ambulatory Controls per Sex
eTable 8. Outcomes for Patients Who Used Wheelchairs vs Matched Ambulatory Controls per Age Group
eTable 9. Outcomes for Patients Who Used Wheelchairs vs Matched Ambulatory Controls per Inclusion Site
eFigure 2. Cumulative Incidence Function in Patients Who Used Wheelchairs vs Matched Ambulatory Controls
eTable 10. Fine and Gray–Adjusted Subdistribution Hazard Ratios for Patients Who Used Wheelchairs vs Matched Ambulatory Controls With Consideration of Competing Risk of Death

This supplementary material has been provided by the authors to give readers additional information about their work.

© 2023 Axelsson KF et al. *JAMA Network Open*.

eMethods. Additional Information on Registers Used and Standardized Mean Difference

Additional Information on Registers used

National Patient Register: Started in 1964 with diagnoses from admitted patients which became compulsory nationally in 1987. Diagnoses from outpatient visits were added in 2001. Only diagnoses set by physicians are included. We only used ICD-10 codes, implemented which has been registered since 1998.

Swedish Prescribed Drug Register: Started July 1st 2005 and includes ATC-codes of prescribed and collected medications.

Standardized Mean Difference (SMD)

For categorical variables, standardized mean differences were calculated as follows:
https://support.sas.com/resources/papers/proceedings12/335-2012.pdf

Otherwise, standardized mean differences were calculated as follows:

$$SMD = |\mu_{cases} - \mu_{controls}| / [(\sigma_{cases}^2 + \sigma_{controls}^2) / 2]$$

© 2023 Axelsson KF et al. *JAMA Network Open*.

eFigure 1. Study Population

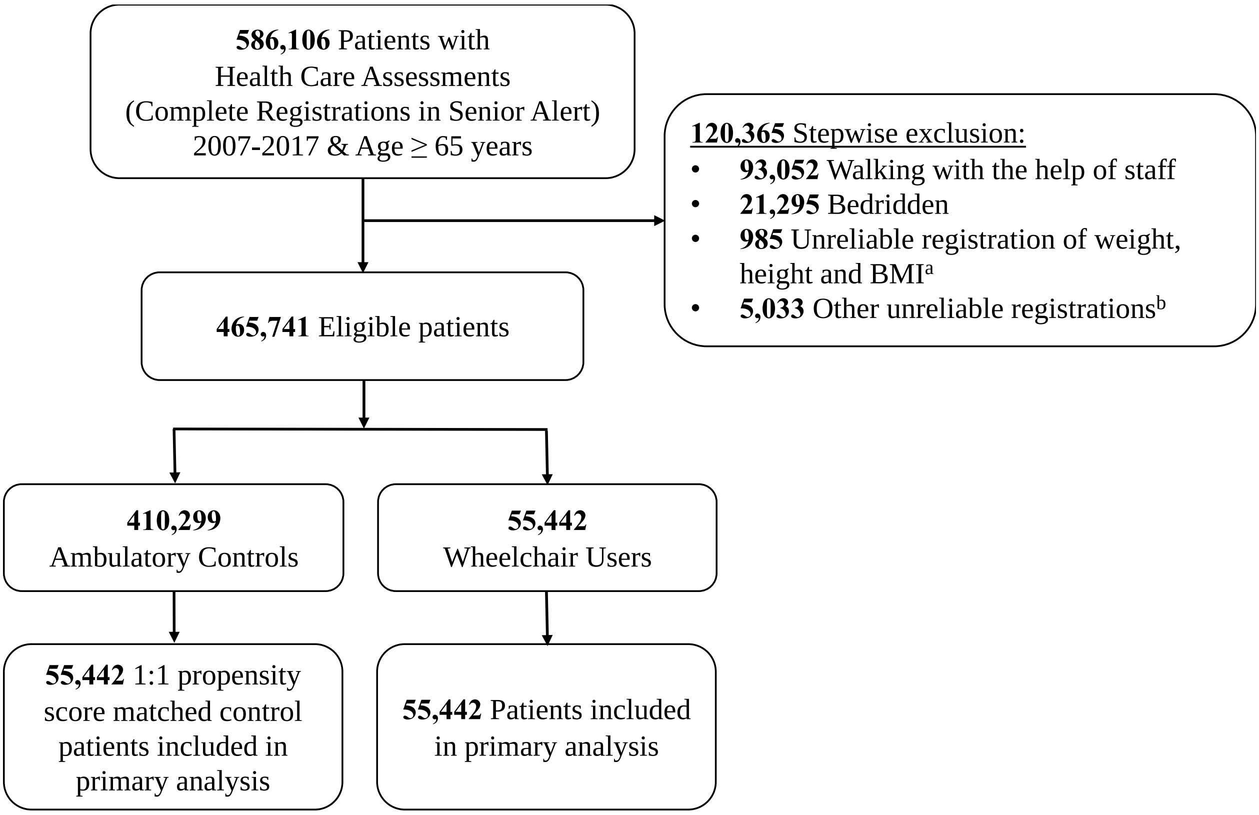

a. Accepted values after exclusion of top and bottom 0.1% of weight, height, and body mass index (BMI)
b. Faulty mortality data, not residing in Sweden at study inclusion, invalid fracture dates

© 2023 Axelsson KF et al. *JAMA Network Open*.

eTable 1. Detailed Definitions of Outcomes

Variable	Definitions Using ICD-10 Codes
Any Fracture	S12, S22, S32, S42, S52, S62 (excluding fingers S625-S627), S72, S82, S92 (excluding toes S924-S925), T02, T08, T10, T12, T142, M485
Major Osteoporotic Fracture	hip (S720-S722), wrist (S525-S526), collum chirugicum (S422), vertebra (S220, S221, S320), pelvis (S32)
Vertebral fracture	S220, S221, S320
Hip Fracture	S720-S722 with a code for surgical procedure (NFB, NFC, NFJ)
Distal femoral fracture	S724
Proximal tibia fracture	S821
Ankle fracture	S825, S826, S828
Proximal Humerus Fracture	S422
Wrist Fracture	S525, S526
Injurious fall	W00-W19 code and a S00-T14 diagnosis, but not a simultaneous fracture code

Fracture data was refined in multiple steps. First, fracture diagnoses with a simultaneous code indicating a revisit (Z09, Z47, Z48) and hip fracture diagnoses without a simultaneous code for surgical procedure were discarded. Second, a washout period of five months was used, so that if a fracture diagnosis referring to the same skeletal site was repeated within a period of five months, the latter diagnosis was discarded to avoid including codes from revisits. Incident hip fracture included fractures of the femoral head, neck, trochanter or subtrochanteric part of the femur accompanied with a code for surgical procedure (NFB, NFC or NFJ).

© 2023 Axelsson KF et al. *JAMA Network Open*.

eTable 2. Detailed Definitions of Selected Covariates

Variable	Definitions Using ICD-10 Codes and/or ATC-Codes
Age	At baseline
Sickness benefits	Any payment from the social services during the baseline year
Marital status	Categorized as married, unmarried, divorced or widowed. Registered partnerships considered as marriages in respective category. Value taken from end of the baseline year, if missing the adjacent years are used if available.
Urban residency, (>200 per km^2)	The municipal registration at baseline is used and its population density
Non-Nordic citizenship at birth	Other citizenships at birth than Swedish, Norwegian, Danish, Finish or Icelandic
Charlson comorbidity index (weight)	The weighted sum of the diseases below. Diagnoses from both outpatient visits and admissions used. NOTE: Diabetes not included.
Dementia (1)	F00-F03
Ischemic heart disease (1)	I20-I25
Heart failure (1)	I50
Cerebrovascular disease (1)	I60-I69
Vascular diseases (1)	I70-I79
Chronic pulmonary diseases (1)	J43-J46
Chronic liver disease (1)	K70-K77
Tumor without metastasis (2)	C00-C76, C80, C97
Lymphoma or leukemia (2)	C81-C96
Diabetes (1)	E10-E14
with end organ damage (+1)	E102, E103, E104, E105, E107, E112, E113, E114, E115, E117, H360
Kidney disease (1)	N17-N19
moderate or severe (+1)	N18.2-N18.5
Hemiplegia (2)	G81
Peptic ulcer disease (2)	K25-K27
Metastatic solid tumor (6)	C77-C79
Osteoporosis diagnosis	M80-M81
Conditions associated with osteoporosis	Hyperthyroidism (E05), Hypogonadism (E28-E29), Malnutrition (E40-E46), Osteogenesis imperfecta (Q780), Chronic liver disease (K70-K77), Hyperparathyroidism (E21)
Alcohol related disease	Mental and behavioral disorders due to use of alcohol (F10), Degeneration of nervous system due to alcohol (G312), Alcoholic polyneuropathy (G621), Alcoholic myopathy (G721), Alcoholic cardiomyopathy (I426), Alcoholic gastritis (K292), Alcoholic liver disease (K70), Alcohol-induced acute pancreatitis (K852), Alcohol-induced chronic pancreatitis (K860), Toxic effect of alcohol (T51)
Rheumatoid arthritis	M05-M06
Osteoporosis medication	Prescriptions: Bisphosphonates (M05BA and M05BB), Denosumab (M05BX04), Teriparatide (H05AA02), Strontium (M05BX03), Raloxifene (G03XC01), Testosterone (G03BA03), Systemic estrogens (G03CA) Tibolone (G03CX01).<br><br>Non-prescribed parenteral treatment offered to patients at outpatient clinics or while admitted: The combination of an osteoporosis diagnosis (M80, M81 or M859) and a code for intravenous (DT016) or subcutaneous (DT021) administration was used.
Calcium + Vitamin D	ATC-code A12AX, repeated (≥2 prescriptions last year) and recent (last prescription within 120 days)
Oral prednisolone	Any previous three-month period in which more than 450 mg prednisolone (ATC-code H02AB06) were collected, i.e. more than 5 mg/day within a three-month period
Prevalent fracture	Any previous fracture (see outcomes for definitions)
Prevalent fall injury	Any previous non-skeletal fall injury resulting in a hospital visit or admission. W00-W19 code and a S00-T14 diagnosis, but not a simultaneous fracture code.

© 2023 Axelsson KF et al. *JAMA Network Open*.

eTable 3. Baseline Characteristics of Variables Possibly Associated with Wheelchair Use

Variable*	Wheelchair Users n=55,442	Ambulatory Controls Before matching n=410,299	SMD[a]	Ambulatory Controls After matching n=55,442	SMD[a]
Previous stroke, n (%)	22,354 (40.3)	59,308 (14.5)	0.606	10,179 (18.4)	0.497
Previous femur fracture, n (%)	13,552 (24.4)	31,271 (7.6)	0.471	8,956 (16.2)	0.207
Previous lower leg fracture, n (%)	5,104 (9.2)	18,690 (4.6)	0.185	3,475 (6.3)	0.110
Hemiplegia, n (%)	5,047 (9.1)	4,696 (1.1)	0.367	877 (1.6)	0.339
Para- or tetraplegia, n (%)	990 (1.8)	388 (0.1)	0.176	62 (0.1)	0.173
Epilepsy, n (%)	3,408 (6.1)	8,246 (2.0)	0.210	1,600 (2.9)	0.158
Parkinson, n (%)	2,995 (5.4)	7,347 (1.8)	0.195	1,404 (2.5)	0.147
Spinal cord injury, n (%)	270 (0.5)	544 (0.1)	0.064	79 (0.1)	0.062

Baseline characteristics of variables possibly associated with wheelchair use, and therefor not included in the matching. Presented for wheelchair users vs ambulatory controls, before and after propensity score matching
*Variables included previous stroke (I61, I63, I64, I69), previous femur fracture (S72), previous lower leg fracture (S82), hemiplegia (G81), para- or tetraplegia (G82), parkinson (G20-G21), epilepsy (G40), spinal cord injury (S14, S24, S34.

© 2023 Axelsson KF et al. *JAMA Network Open*.

eTable 4. Other Outcomes for Patients Who Used Wheelchairs vs Matched Ambulatory Controls

	**Ambulatory Controls** n=55,442	**Wheelchair Users** n=55,442
Time at risk, years median (IQR)	2.3 (0.75-3.6)	2.0 (0.54-3.2)
**Ankle Fracture**		
n (%)	373 (0.67%)	316 (0.57%)
Rate, per 1000 person-years	2.90 (2.61-3.21)	2.81 (2.51-3.13)
Cox, unadjusted, HR (95%CI)	Ref. [1]	0.96 (0.83-1.12)
Cox, adjusted, HR (95%CI)	Ref. [1]	0.95 (0.82-1.10)
**Proximal Humerus Fracture**		
n (%)	1,063 (1.92%)	397 (0.72%)
Rate, per 1000 person-years	8.32 (7.83-8.84)	3.53 (3.19-3.89)
Cox, unadjusted, HR (95%CI)	Ref. [1]	0.42 (0.38-0.47)
Cox, adjusted, HR (95%CI)	Ref. [1]	0.43 (0.38-0.48)
**Wrist Fracture**		
n (%)	1,172 (2.11%)	237 (0.43%)
Rate, per 1000 person-years	9.21 (8.69-9.76)	2.10 (1.84-2.39)
Cox, unadjusted, HR (95%CI)	Ref. [1]	0.23 (0.20-0.26)
Cox, adjusted, HR (95%CI)	Ref. [1]	0.23 (0.20-0.27)

Outcomes for wheelchair users and propensity score matched ambulatory controls. Event rates were calculated as the number of events per 1000 person-years and are presented with exact Poisson 95% confidence intervals. The adjusted Cox model is adjusted for age, sex, weight, height, patient place at time of registration, general condition, fluid intake, food intake, sickness benefits, marital status, urban residency, non-Nordic citizenship at birth, Charlson comorbidity index, osteoporosis diagnosis, conditions associated with osteoporosis, alcohol related disease, rheumatoid arthritis, prevalent fracture, prevalent fall injury, osteoporosis medication, calcium + vitamin D and oral prednisolone.

© 2023 Axelsson KF et al. *JAMA Network Open*.

eTable 5. Outcomes for Patients Who Used Wheelchairs vs Unmatched Ambulatory Controls

	**Ambulatory Unmatched Controls** n=410,299	**Wheelchair Users** n=55,442
Time at risk, years median (IQR)	2.9 (1.2-4.4)	2 (0.54-3.2)
**Any Fracture**		
n (%)	63,781 (15.5%)	4,148 (7.48%)
Rate, per 1000 person-years	58.2 (57.8-58.7)	39.3 (38.1-40.5)
Cox, unadjusted, HR (95%CI)	Ref. [1]	0.66 (0.64-0.68)
Cox, adjusted, HR (95%CI)	Ref. [1]	0.44 (0.42-0.45)
**Major Osteoporotic Fracture**		
n (%)	47,286 (11.5%)	2,399 (4.33%)
Rate, per 1000 person-years	41.9 (41.5-42.3)	22.0 (21.2-22.9)
Cox, unadjusted, HR (95%CI)	Ref. [1]	0.51 (0.49-0.53)
Cox, adjusted, HR (95%CI)	Ref. [1]	0.33 (0.31-0.34)
**Vertebral Fracture**		
n (%)	3,400 (0.829%)	157 (0.283%)
Rate, per 1000 person-years	2.83 (2.73-2.92)	1.39 (1.18-1.62)
Cox, unadjusted, HR (95%CI)	Ref. [1]	0.48 (0.41-0.57)
Cox, adjusted, HR (95%CI)	Ref. [1]	0.44 (0.37-0.52)
**Hip Fracture**		
n (%)	26,411 (6.44%)	1,363 (2.46%)
Rate, per 1000 person-years	22.6 (22.3-22.9)	12.3 (11.7-13.0)
Cox, unadjusted, HR (95%CI)	Ref. [1]	0.53 (0.50-0.56)
Cox, adjusted, HR (95%CI)	Ref. [1]	0.31 (0.29-0.33)
**Distal Femur Fracture**		
n (%)	868 (0.212%)	327 (0.590%)
Rate, per 1000 person-years	0.718 (0.671-0.768)	2.90 (2.59-3.23)
Cox, unadjusted, HR (95%CI)	Ref. [1]	4.12 (3.62-4.68)
Cox, adjusted, HR (95%CI)	Ref. [1]	2.78 (2.41-3.20)
**Proximal Tibia Fracture**		
n (%)	957 (0.233%)	205 (0.370%)
Rate, per 1000 person-years	0.792 (0.743-0.844)	1.82 (1.58-2.08)
Cox, unadjusted, HR (95%CI)	Ref. [1]	2.30 (1.98-2.68)
Cox, adjusted, HR (95%CI)	Ref. [1]	1.70 (1.45-2.01)
**Fall Injury w/o Fracture**		
n (%)	52,926 (12.9%)	3,544 (6.39%)
Rate, per 1000 person-years	47.4 (47.0-47.8)	33.1 (32.0-34.2)
Cox, unadjusted, HR (95%CI)	Ref. [1]	0.68 (0.66-0.70)
Cox, adjusted, HR (95%CI)	Ref. [1]	0.50 (0.48-0.52)
**Death**		
n (%)	175,912 (42.9%)	40,722 (73.4%)
Rate, per 1000 person-years	145 (145-146)	360 (356-363)
Cox, unadjusted, HR (95%CI)	Ref. [1]	2.41 (2.39-2.44)
Cox, adjusted, HR (95%CI)	Ref. [1]	1.38 (1.37-1.40)

Outcomes for wheelchair users and propensity score matched ambulatory controls. Event rates were calculated as the number of events per 1000 person-years and are presented with exact Poisson 95% confidence intervals. The adjusted Cox model is adjusted for age, sex, weight, height, patient place at time of registration, general condition, fluid intake, food intake, sickness benefits, marital status, urban residency, non-Nordic citizenship at birth, Charlson comorbidity index, osteoporosis diagnosis, conditions associated with osteoporosis, alcohol related disease, rheumatoid arthritis, prevalent fracture, prevalent fall injury, osteoporosis medication, calcium + vitamin D and oral prednisolone. Fall Injury w/o Fracture = Injurious falls without Fracture.All p-values <0.001.

© 2023 Axelsson KF et al. *JAMA Network Open*.

eTable 6. Outcomes for Patients Who Used Wheelchairs vs Matched Ambulatory Controls per Subgroup

	Ambulatory Controls	Wheelchair Users and SCI	para/tetra	Parkinson	Epilepsy	Hemiplegia	Lower leg Fracture	Femur Fracture	Stroke	No Known Cause
N=	55442	270	826	2967	3162	4028	4078	9931	11438	18742
Time at risk - years median (IQR)	2.3 (0.75-3.6)	2.6 (0.72-4)	2.5 (0.84-3.8)	1.8 (0.55-2.6)	2.2 (0.65-3.4)	2.3 (0.67-3.5)	2.4 (0.78-3.7)	1.9 (0.5-2.9)	2.2 (0.6-3.4)	1.9 (0.43-2.9)
**Any Fracture**										
n (%)	10,344 (18.7%)	31 (11.5%)	65 (7.87%)	164 (5.53%)	256 (8.1%)	300 (7.45%)	591 (14.5%)	795 (8.01%)	755 (6.6%)	1,191 (6.35%)
Rate, per 1000 pys	91.2 (89.4-93.0)	49.1 (33.4-69.8)	34.0 (26.2-43.3)	32.4 (27.7-37.8)	39.3 (34.7-44.5)	34.9 (31.1-39.1)	70.2 (64.7-76.1)	45.9 (42.7-49.2)	31.6 (29.4-34.0)	35.7 (33.7-37.8)
Cox, unadj., HR (95%CI)	Ref. [1]	0.55 (0.39-0.78)	0.38 (0.30-0.48)	0.34 (0.30-0.40)	0.43 (0.38-0.49)	0.39 (0.34-0.43)	0.77 (0.71-0.84)	0.49 (0.46-0.53)	0.35 (0.32-0.38)	0.39 (0.36-0.41)
Cox, adj, HR (95%CI)	Ref. [1]	0.57 (0.40-0.81)	0.47 (0.37-0.60)	0.35 (0.30-0.41)	0.47 (0.42-0.54)	0.42 (0.38-0.48)	0.65 (0.59-0.70)	0.37 (0.34-0.39)	0.42 (0.39-0.45)	0.43 (0.40-0.46)
**MOF**										
n (%)	8,046 (14.5%)	15 (5.56%)	21 (2.54%)	93 (3.13%)	132 (4.17%)	187 (4.64%)	298 (7.31%)	419 (4.22%)	504 (4.41%)	730 (3.89%)
Rate, per 1000 pys	68.5 (67.0-70.0)	22.8 (12.8-37.6)	10.5 (6.51-16.1)	18.0 (14.5-22.0)	19.6 (16.4-23.2)	21.2 (18.3-24.5)	32.9 (29.2-36.8)	23.4 (21.2-25.7)	20.7 (19.0-22.6)	21.4 (19.9-23.0)
Cox, unadj., HR (95%CI)	Ref. [1]	0.34 (0.21-0.57)	0.16 (0.10-0.24)	0.25 (0.21-0.31)	0.29 (0.24-0.34)	0.31 (0.27-0.36)	0.48 (0.43-0.54)	0.33 (0.30-0.37)	0.30 (0.28-0.33)	0.31 (0.29-0.33)
Cox, adj, HR (95%CI)	Ref. [1]	0.39 (0.24-0.65)	0.21 (0.14-0.32)	0.26 (0.21-0.32)	0.33 (0.27-0.39)	0.35 (0.30-0.41)	0.43 (0.39-0.49)	0.25 (0.22-0.27)	0.36 (0.33-0.40)	0.34 (0.31-0.36)
**Hip Fracture**										
n (%)	4,971 (8.97%)	6 (2.22%)	13 (1.57%)	58 (1.95%)	60 (1.9%)	105 (2.61%)	146 (3.58%)	199 (2%)	337 (2.95%)	439 (2.34%)
Rate, per 1000 pys	40.5 (39.4-41.7)	8.88 (3.26-19.3)	6.46 (3.44-11.0)	11.0 (8.39-14.3)	8.68 (6.63-11.2)	11.7 (9.57-14.2)	15.6 (13.1-18.3)	10.9 (9.41-12.5)	13.7 (12.2-15.2)	12.7 (11.5-13.9)
Cox, unadj., HR (95%CI)	Ref. [1]	0.22 (0.10-0.50)	0.16 (0.09-0.28)	0.26 (0.20-0.34)	0.22 (0.17-0.28)	0.29 (0.24-0.35)	0.39 (0.33-0.46)	0.26 (0.23-0.30)	0.34 (0.30-0.38)	0.31 (0.28-0.34)
Cox, adj, HR (95%CI)	Ref. [1]	0.29 (0.13-0.64)	0.22 (0.13-0.38)	0.27 (0.21-0.35)	0.25 (0.19-0.32)	0.33 (0.27-0.40)	0.39 (0.33-0.46)	0.20 (0.17-0.23)	0.39 (0.35-0.43)	0.33 (0.30-0.36)

*Abbreviations used: pys=person-years. Unadj.=unadjusted. Adj.=Adjusted.
Outcomes for wheelchair users and propensity score matched ambulatory controls. Event rates were calculated as the number of events per 1000 person-years and are presented with exact Poisson 95% confidence intervals. The adjusted Cox model is adjusted for age, sex, weight, height, patient place at time of registration, general condition, fluid intake, food intake, sickness benefits, marital status, urban residency, non-Nordic citizenship at birth, Charlson comorbidity index, osteoporosis diagnosis, conditions associated with osteoporosis, alcohol related disease, rheumatoid arthritis, prevalent fracture, prevalent fall injury, osteoporosis medication, calcium + vitamin D and oral prednisolone. All p-values <0.001.

© 2023 Axelsson KF et al. *JAMA Network Open*.

eTable 7. Outcomes for Patients Who Used Wheelchairs vs Matched Ambulatory Controls per Sex

	All		Males		Females	
	**Ambulatory Controls**	**Wheelchair Users**	**Ambulatory Controls**	**Wheelchair Users**	**Ambulatory Controls**	**Wheelchair Users**
**N=**	**55442**	**55442**	**21736**	**21895**	**33706**	**33547**
Time at risk, years median (IQR)	2.3 (0.75-3.6)	2 (0.54-3.2)	2.2 (0.62-3.4)	2 (0.47-3)	2.4 (0.86-3.7)	2.1 (0.58-3.2)
**Any Fracture**						
n (%)	10,344 (18.7%)	4,148 (7.48%)	2,903 (13.4%)	1,327 (6.06%)	7,441 (22.1%)	2,821 (8.41%)
Rate, per 1000 person-years	91.2 (89.4-93.0)	39.3 (38.1-40.5)	66.5 (64.1-69.0)	32.4 (30.7-34.2)	107 (104-109)	43.6 (42.0-45.2)
Cox, unadjusted, HR (95%CI)	Ref. [1]	0.43 (0.41-0.44)	Ref. [1]	0.48 (0.45-0.52)	Ref. [1]	0.41 (0.39-0.42)
Cox, adjusted, HR (95%CI)	Ref. [1]	0.43 (0.42-0.45)	Ref. [1]	0.48 (0.45-0.51)	Ref. [1]	0.41 (0.40-0.43)
**Major Osteoporotic Fracture**						
n (%)	8,046 (14.5%)	2,399 (4.33%)	2,200 (10.1%)	816 (3.73%)	5,846 (17.3%)	1,583 (4.72%)
Rate, per 1000 person-years	68.5 (67.0-70.0)	22.0 (21.2-22.9)	49.1 (47.1-51.2)	19.5 (18.2-20.9)	80.5 (78.4-82.6)	23.6 (22.5-24.8)
Cox, unadjusted, HR (95%CI)	Ref. [1]	0.32 (0.31-0.33)	Ref. [1]	0.39 (0.36-0.43)	Ref. [1]	0.29 (0.28-0.31)
Cox, adjusted, HR (95%CI)	Ref. [1]	0.33 (0.31-0.34)	Ref. [1]	0.39 (0.36-0.43)	Ref. [1]	0.30 (0.28-0.32)
**Hip Fracture**						
n (%)	4,971 (8.97%)	1,363 (2.46%)	1,431 (6.58%)	517 (2.36%)	3,540 (10.5%)	846 (2.52%)
Rate, per 1000 person-years	40.5 (39.4-41.7)	12.3 (11.7-13.0)	31.2 (29.6-32.8)	12.2 (11.2-13.3)	46.1 (44.6-47.6)	12.4 (11.5-13.2)
Cox, unadjusted, HR (95%CI)	Ref. [1]	0.30 (0.28-0.32)	Ref. [1]	0.39 (0.35-0.43)	Ref. [1]	0.27 (0.25-0.29)
Cox, adjusted, HR (95%CI)	Ref. [1]	0.31 (0.29-0.33)	Ref. [1]	0.39 (0.35-0.43)	Ref. [1]	0.27 (0.25-0.29)

© 2023 Axelsson KF et al. *JAMA Network Open*.

eTable 8. Outcomes for Patients Who Used Wheelchairs vs Matched Ambulatory Controls per Age Group

	All		Age 65-79		Age 80-87		Age 88+	
	**Ambulatory Controls**	**Wheelchair Users**	**Ambulatory Controls**	**Wheelchair Users**	**Ambulatory Controls**	**Wheelchair Users**	**Ambulatory Controls**	**Wheelchair Users**
**N=**	**55442**	**55442**	**17050**	**17647**	**19086**	**18931**	**19306**	**18864**
Time at risk, years median (IQR)	2.3 (0.75-3.6)	2 (0.54-3.2)	2.7 (0.86-4.2)	2.6 (0.8-4.1)	2.4 (0.82-3.7)	2 (0.57-3.1)	1.9 (0.65-2.9)	1.5 (0.38-2.2)
**Any Fracture**								
n (%)	10,344 (18.7%)	4,148 (7.48%)	2,341 (13.7%)	1,641 (9.30%)	3,959 (20.7%)	1,421 (7.51%)	4,044 (20.9%)	1,086 (5.76%)
Rate, per 1000 person-years	91.2 (89.4-93.0)	39.3 (38.1-40.5)	56.4 (54.2-58.8)	38.7 (36.9-40.7)	99.8 (96.8-103)	39.4 (37.4-41.5)	125 (121-129)	39.9 (37.6-42.4)
Cox, unadjusted, HR (95%CI)	Ref. [1]	0.43 (0.41-0.44)	Ref. [1]	0.69 (0.64-0.73)	Ref. [1]	0.39 (0.37-0.42)	Ref. [1]	0.31 (0.29-0.34)
Cox, adjusted, HR (95%CI)	Ref. [1]	0.43 (0.42-0.45)	Ref. [1]	0.68 (0.63-0.72)	Ref. [1]	0.39 (0.37-0.42)	Ref. [1]	0.31 (0.29-0.33)
**Major Osteoporotic Fracture**								
n (%)	8,046 (14.5%)	2,399 (4.33%)	1,646 (9.65%)	889 (5.04%)	3,131 (16.4%)	892 (4.71%)	3,269 (16.9%)	618 (3.28%)
Rate, per 1000 person-years	68.5 (67.0-70.0)	22.0 (21.2-22.9)	38.4 (36.5-40.2)	20.2 (18.9-21.6)	76.2 (73.6-78.9)	24.1 (22.5-25.7)	97.8 (94.5-101)	22.2 (20.5-24.1)
Cox, unadjusted, HR (95%CI)	Ref. [1]	0.32 (0.31-0.33)	Ref. [1]	0.53 (0.49-0.57)	Ref. [1]	0.31 (0.29-0.34)	Ref. [1]	0.22 (0.21-0.24)
Cox, adjusted, HR (95%CI)	Ref. [1]	0.33 (0.31-0.34)	Ref. [1]	0.52 (0.48-0.56)	Ref. [1]	0.31 (0.29-0.34)	Ref. [1]	0.22 (0.20-0.24)
**Hip Fracture**								
n (%)	4,971 (8.97%)	1,363 (2.46%)	919 (5.39%)	475 (2.69%)	1,974 (10.3%)	536 (2.83%)	2,078 (10.8%)	352 (1.87%)
Rate, per 1000 person-years	40.5 (39.4-41.7)	12.3 (11.7-13.0)	20.7 (19.4-22.1)	10.6 (9.64-11.6)	45.7 (43.7-47.7)	14.2 (13.0-15.5)	59.2 (56.7-61.8)	12.5 (11.2-13.9)
Cox, unadjusted, HR (95%CI)	Ref. [1]	0.30 (0.28-0.32)	Ref. [1]	0.51 (0.46-0.57)	Ref. [1]	0.31 (0.28-0.34)	Ref. [1]	0.21 (0.18-0.23)
Cox, adjusted, HR (95%CI)	Ref. [1]	0.31 (0.29-0.33)	Ref. [1]	0.49 (0.44-0.55)	Ref. [1]	0.31 (0.28-0.34)	Ref. [1]	0.21 (0.18-0.23)
**Death**								
n (%)	34,279 (61.8%)	40,722 (73.4%)	7,667 (45.0%)	10,049 (56.9%)	12,086 (63.3%)	14,402 (76.1%)	14,526 (75.2%)	16,271 (86.3%)
Rate, per 1000 person-years	265 (262-268)	360 (356-363)	167 (164-171)	219 (215-223)	263 (258-267)	373 (367-379)	387 (380-393)	567 (558-576)
Cox, unadjusted, HR (95%CI)	Ref. [1]	1.35 (1.33-1.36)	Ref. [1]	1.30 (1.26-1.34)	Ref. [1]	1.41 (1.38-1.45)	Ref. [1]	1.47 (1.43-1.50)
Cox, adjusted, HR (95%CI)	Ref. [1]	1.40 (1.38-1.42)	Ref. [1]	1.30 (1.26-1.34)	Ref. [1]	1.42 (1.38-1.45)	Ref. [1]	1.46 (1.42-1.49)

Outcomes for wheelchair users and propensity score matched ambulatory controls. Event rates were calculated as the number of events per 1000 person-years and are presented with exact Poisson 95% confidence intervals. The adjusted Cox model is adjusted for age, sex, weight, height, patient place at time of registration, general condition, fluid intake, food intake, sickness benefits, marital status, urban residency, non-Nordic citizenship at birth, Charlson comorbidity index, osteoporosis diagnosis, conditions associated with osteoporosis, alcohol related disease, rheumatoid arthritis, prevalent fracture, prevalent fall injury, osteoporosis medication, calcium + vitamin D and oral prednisolone. All p-values <0.001.

© 2023 Axelsson KF et al. *JAMA Network Open*.

eTable 9. Outcomes for Patients Who Used Wheelchairs vs Matched Ambulatory Controls per Inclusion Site

	**All**		**Nursing home**		**Hospital**		**Private residence**	
	**Ambulatory Controls**	**Wheelchair Users**	**Ambulatory Controls**	**Wheelchair Users**	**Ambulatory Controls**	**Wheelchair Users**	**Ambulatory Controls**	**Wheelchair Users**
**N=**	**55442**	**55442**	**34814**	**34773**	**17174**	**17354**	**3454**	**3315**
Time at risk, years median (IQR)	2.3 (0.75-3.6)	2 (0.54-3.2)	2.1 (0.73-3.2)	1.8 (0.52-2.7)	2.7 (0.82-4.2)	2.4 (0.53-3.8)	2.4 (0.74-3.7)	2.4 (0.81-3.8)
**Any Fracture**								
n (%)	10,344 (18.7%)	4,148 (7.48%)	7,140 (20.5%)	1,700 (4.89%)	2,515 (14.6%)	2,114 (12.2%)	689 (19.9%)	334 (10.1%)
Rate, per 1000 person-years	91.2 (89.4-93.0)	39.3 (38.1-40.5)	111 (108-113)	27.7 (26.4-29.1)	60.0 (57.7-62.4)	57.4 (55.0-59.9)	98.1 (90.9-106)	44.8 (40.1-49.9)
Cox, unadjusted, HR (95%CI)	Ref. [1]	0.43 (0.41-0.44)	Ref. [1]	0.25 (0.24-0.26)	Ref. [1]	0.95 (0.89-1.00)	Ref. [1]	0.46 (0.40-0.52)
Cox, adjusted, HR (95%CI)	Ref. [1]	0.43 (0.42-0.45)	Ref. [1]	0.25 (0.24-0.27)	Ref. [1]	0.88 (0.83-0.93)	Ref. [1]	0.52 (0.46-0.60)
**Major Osteoporotic Fracture**								
n (%)	8,046 (14.5%)	2,399 (4.33%)	5,764 (16.6%)	907 (2.61%)	1,779 (10.4%)	1,321 (7.61%)	503 (14.6%)	171 (5.16%)
Rate, per 1000 person-years	68.5 (67.0-70.0)	22.0 (21.2-22.9)	86.4 (84.1-88.6)	14.5 (13.6-15.5)	41.1 (39.2-43.0)	34.4 (32.5-36.3)	68.2 (62.3-74.4)	22.1 (18.9-25.7)
Cox, unadjusted, HR (95%CI)	Ref. [1]	0.32 (0.31-0.33)	Ref. [1]	0.17 (0.16-0.18)	Ref. [1]	0.83 (0.77-0.89)	Ref. [1]	0.33 (0.27-0.39)
Cox, adjusted, HR (95%CI)	Ref. [1]	0.33 (0.31-0.34)	Ref. [1]	0.17 (0.16-0.18)	Ref. [1]	0.76 (0.71-0.82)	Ref. [1]	0.39 (0.32-0.46)
**Hip Fracture**								
n (%)	4,971 (8.97%)	1,363 (2.46%)	3,739 (10.7%)	541 (1.56%)	939 (5.47%)	724 (4.17%)	293 (8.48%)	98 (2.96%)
Rate, per 1000 person-years	40.5 (39.4-41.7)	12.3 (11.7-13.0)	53.4 (51.7-55.2)	8.55 (7.85-9.31)	20.9 (19.6-22.3)	18.2 (16.9-19.6)	37.8 (33.6-42.4)	12.4 (10.1-15.1)
Cox, unadjusted, HR (95%CI)	Ref. [1]	0.30 (0.28-0.32)	Ref. [1]	0.16 (0.14-0.17)	Ref. [1]	0.86 (0.78-0.95)	Ref. [1]	0.33 (0.26-0.41)
Cox, adjusted, HR (95%CI)	Ref. [1]	0.31 (0.29-0.33)	Ref. [1]	0.16 (0.15-0.18)	Ref. [1]	0.80 (0.72-0.88)	Ref. [1]	0.39 (0.31-0.49)
**Death**								
n (%)	34,279 (61.8%)	40,722 (73.4%)	23,967 (68.8%)	27,609 (79.4%)	8,249 (48.0%)	11,052 (63.7%)	2,063 (59.7%)	2,061 (62.2%)
Rate, per 1000 person-years	265 (262-268)	360 (356-363)	320 (316-324)	431 (426-436)	178 (174-182)	269 (264-274)	252 (241-263)	256 (245-267)
Cox, unadjusted, HR (95%CI)	Ref. [1]	1.35 (1.33-1.36)	Ref. [1]	1.35 (1.32-1.37)	Ref. [1]	1.48 (1.44-1.52)	Ref. [1]	1.01 (0.95-1.08)
Cox, adjusted, HR (95%CI)	Ref. [1]	1.40 (1.38-1.42)	Ref. [1]	1.40 (1.37-1.42)	Ref. [1]	1.46 (1.42-1.50)	Ref. [1]	1.30 (1.22-1.38)

Outcomes for wheelchair users and propensity score matched ambulatory controls per inclusion site. Event rates were calculated as the number of events per 1000 person-years and are presented with exact Poisson 95% confidence intervals. The adjusted Cox model is adjusted for age, sex, weight, height, general condition, fluid intake, food intake, sickness benefits, marital status, urban residency, non-Nordic citizenship at birth, Charlson comorbidity index, osteoporosis diagnosis, conditions associated with osteoporosis, alcohol related disease, rheumatoid arthritis, prevalent fracture, prevalent fall injury, osteoporosis medication, calcium + vitamin D and oral prednisolone. All p-values <0.001.

© 2023 Axelsson KF et al. *JAMA Network Open*.

eFigure 2. Cumulative Incidence Function in Patients Who Used Wheelchairs vs Matched Ambulatory Controls

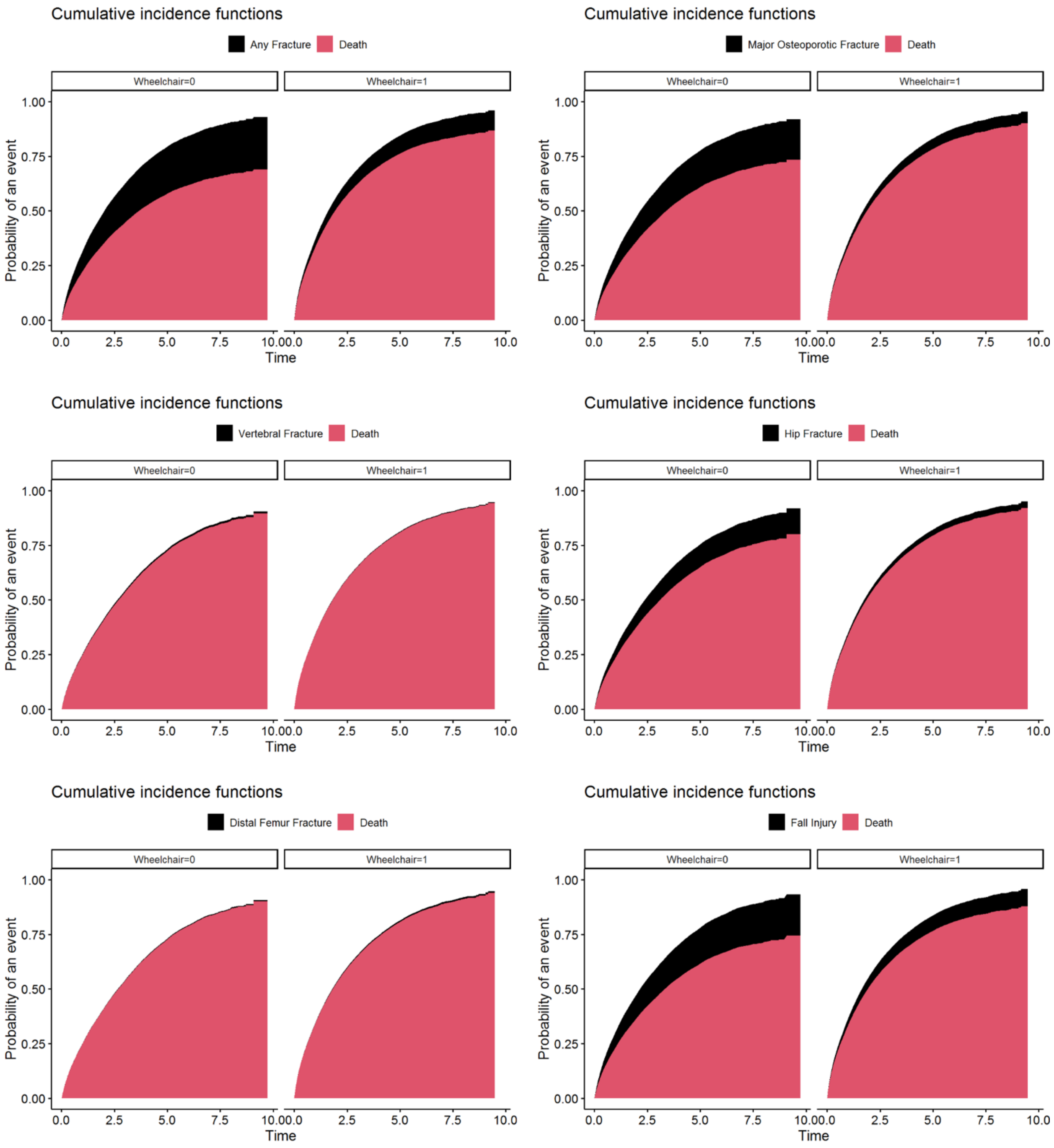

Cumulative Incidence Function in Wheelchair Users vs Ambulatory Controls. The cumulative incidence function, or subdistribution function, of fracture/injurious fall with death as competing risk was estimated using the Aalen-Johansen estimator. All patients included.

© 2023 Axelsson KF et al. *JAMA Network Open*.

eTable 10. Fine and Grey Adjusted Subdistribution Hazard Ratios for Patients Who Used Wheelchairs vs. Matched Ambulatory Controls with Consideration of Competing Risk of Death

Age group	Event	SHR (95% CI)	p-value	HR (95% CI)
65-79	Any Fracture	0.62 (0.58-0.66)	<0.001	0.68 (0.63-0.72)
65-79	Major Osteoporotic Fracture	0.47 (0.44-0.51)	<0.001	0.52 (0.48-0.56)
65-79	Vertebral Fracture	0.71 (0.53-0.95)	0.02	0.77 (0.57-1.03)
65-79	Hip Fracture	0.45 (0.40-0.51)	<0.001	0.49 (0.44-0.55)
65-79	Distal Femur Fracture	2.93 (2.01-4.27)	<0.001	3.28 (2.25-4.77)
65-79	Proximal Tibia Fracture	2.08 (1.45-3.00)	<0.001	2.25 (1.56-3.23)
65-79	Ankle Fracture	1.13 (0.89-1.44)	0.31	1.24 (0.98-1.57)
65-79	Proximal Humerus Fracture	0.54 (0.44-0.65)	<0.001	0.59 (0.49-0.71)
65-79	Wrist Fracture	0.31 (0.25-0.40)	<0.001	0.34 (0.27-0.43)
65-79	Fall Injury w/o Fracture	0.62 (0.58-0.67)	<0.001	0.67 (0.63-0.72)
80-87	Any Fracture	0.33 (0.31-0.35)	<0.001	0.39 (0.37-0.42)
80-87	Major Osteoporotic Fracture	0.26 (0.24-0.28)	<0.001	0.31 (0.29-0.34)
80-87	Vertebral Fracture	0.33 (0.24-0.45)	<0.001	0.38 (0.28-0.52)
80-87	Hip Fracture	0.26 (0.23-0.28)	<0.001	0.31 (0.28-0.34)
80-87	Distal Femur Fracture	1.90 (1.35-2.68)	<0.001	2.35 (1.67-3.32)
80-87	Proximal Tibia Fracture	1.21 (0.84-1.72)	0.30	1.44 (1.00-2.08)
80-87	Ankle Fracture	0.83 (0.63-1.09)	0.17	0.98 (0.74-1.29)
80-87	Proximal Humerus Fracture	0.35 (0.29-0.42)	<0.001	0.42 (0.34-0.50)
80-87	Wrist Fracture	0.19 (0.15-0.24)	<0.001	0.23 (0.18-0.29)
80-87	Fall Injury w/o Fracture	0.39 (0.37-0.42)	<0.001	0.46 (0.43-0.50)
≥88	Any Fracture	0.25 (0.23-0.26)	<0.001	0.31 (0.29-0.33)
≥88	Major Osteoporotic Fracture	0.17 (0.16-0.19)	<0.001	0.22 (0.20-0.24)
≥88	Vertebral Fracture	0.19 (0.12-0.28)	<0.001	0.23 (0.15-0.36)
≥88	Hip Fracture	0.16 (0.15-0.18)	<0.001	0.21 (0.18-0.23)
≥88	Distal Femur Fracture	1.72 (1.28-2.31)	<0.001	2.26 (1.68-3.05)
≥88	Proximal Tibia Fracture	0.95 (0.62-1.45)	0.81	1.25 (0.83-1.91)
≥88	Ankle Fracture	0.50 (0.38-0.68)	<0.001	0.64 (0.48-0.86)
≥88	Proximal Humerus Fracture	0.23 (0.18-0.28)	<0.001	0.29 (0.23-0.36)
≥88	Wrist Fracture	0.12 (0.09-0.16)	<0.001	0.15 (0.11-0.20)
≥88	Fall Injury w/o Fracture	0.30 (0.28-0.32)	<0.001	0.38 (0.35-0.41)

Adjusted Subhazard ratios (SHR) with 95% CI for wheelchair users vs ambulatory controls for fractures and injurious falls. Calculated using a Fine & Gray model with death as the competing risk. All ages (≥65) calculated using in a subset of 30.000 randomly selected patients, whereas all patients were included in the age group specific analyses. The SHR were adjusted for age, sex, weight, height, general condition, fluid intake, food intake, sickness benefits, marital status, urban residency, non-Nordic citizenship at birth, Charlson comorbidity index, osteoporosis diagnosis, conditions associated with osteoporosis, alcohol related disease, rheumatoid arthritis, prevalent fracture, prevalent fall injury, osteoporosis medication, calcium + vitamin D and oral prednisolone. Fall Injury w/o Fracture = Injurious falls without Fracture. Corresponding adjusted Hazard Ratios (HR) included shaded in grey for reference.

© 2023 Axelsson KF et al. *JAMA Network Open*.
